# The effects of Medieval dams on genetic divergence and demographic history in brown trout populations

**DOI:** 10.1186/1471-2148-14-122

**Published:** 2014-06-05

**Authors:** Michael M Hansen, Morten T Limborg, Anne-Laure Ferchaud, José-Martin Pujolar

**Affiliations:** 1 Department of Bioscience, Aarhus University, Ny Munkegade 114, DK-8000 Aarhus C, Denmark; 2 School of Aquatic and Fishery Sciences, University of Washington, 1122 NE Boat Street, Box 355020, 98195 Seattle, Washington, USA; 3 National Institute of Aquatic Resources, Technical University of Denmark, Vejlsøvej 39, 8600 Silkeborg, Denmark

**Keywords:** Approximate Bayesian Computation, Divergence time, Effective population size, Habitat fragmentation, Isolation-with-gene-flow model, Microsatellite DNA

## Abstract

**Background:**

Habitat fragmentation has accelerated within the last century, but may have been ongoing over longer time scales. We analyzed the timing and genetic consequences of fragmentation in two isolated lake-dwelling brown trout populations. They are from the same river system (the Gudenå River, Denmark) and have been isolated from downstream anadromous trout by dams established ca. 600–800 years ago. For reference, we included ten other anadromous populations and two hatchery strains. Based on analysis of 44 microsatellite loci we investigated if the lake populations have been naturally genetically differentiated from anadromous trout for thousands of years, or have diverged recently due to the establishment of dams.

**Results:**

Divergence time estimates were based on 1) Approximate Bayesian Computation and 2) a coalescent-based isolation-with-gene-flow model. Both methods suggested divergence times ca. 600–800 years bp, providing strong evidence for establishment of dams in the Medieval as the factor causing divergence. Bayesian cluster analysis showed influence of stocked trout in several reference populations, but not in the focal lake and anadromous populations. Estimates of effective population size using a linkage disequilibrium method ranged from 244 to > 1,000 in all but one anadromous population, but were lower (153 and 252) in the lake populations.

**Conclusions:**

We show that genetic divergence of lake-dwelling trout in two Danish lakes reflects establishment of water mills and impassable dams ca. 600–800 years ago rather than a natural genetic population structure. Although effective population sizes of the two lake populations are not critically low they may ultimately limit response to selection and thereby future adaptation. Our results demonstrate that populations may have been affected by anthropogenic disturbance over longer time scales than normally assumed.

## Background

Nearly all natural ecosystems, and the species that they encompass, experience anthropogenic pressure resulting particularly from habitat destruction, climate change, overharvesting and introduction of exogenous species [1-4]. Land use leading to habitat fragmentation is considered one of the biggest threats to biodiversity [1], also encompassing short- and long-term negative genetic consequences [5-7]. Fragmentation problems are particularly severe in freshwater systems [8], where the one-dimensional structure of river systems causes dams, weirs and other human-made obstructions to represent impassable barriers to many fishes and invertebrates. Accordingly, several studies have documented negative effects of dams on freshwater fish populations that depend on migration between different spawning, nursery and foraging habitats in different parts of their life cycle. In some cases this had led to extirpation of entire populations [9-12]. In other cases fragmentation due to dams has been shown to exert negative genetic effects caused by restricted gene flow and declining population sizes [13-17].

Populations within a species are often distributed across habitats that exhibit different environmental conditions. These differences may lead to variation in local selection regimes and potentially local adaptation [18], the degree of which is determined by the opposing forces of local selection on the one side and gene flow and random genetic drift on the other [19,20]. Whereas the limiting factors of gene flow and drift under pristine conditions are influenced by natural barriers in the landscape and habitat size and quality [21,22], anthropogenic habitat fragmentation leading to decreased gene flow and effective population sizes may substantially shift migration-selection-drift equilibria [23]. The outcome for local adaptation is not trivial [24,25]. Reduced effective population sizes may cause drift to overwhelm directional selection [26] and limit evolutionary potential [27]. Conversely, reduced gene flow may at the same time increase possibilities for local adaption, as directional selection becomes a stronger evolutionary force than immigration [19,28], though in the longer term gene flow into small populations may benefit adaptive responses by introducing new variation. In total, when investigating adaptive divergence between populations in human-altered environments the question arises if local adaption reflects historical or more recent anthropogenically modified selection regimes and demographic parameters.

**Figure 1:**
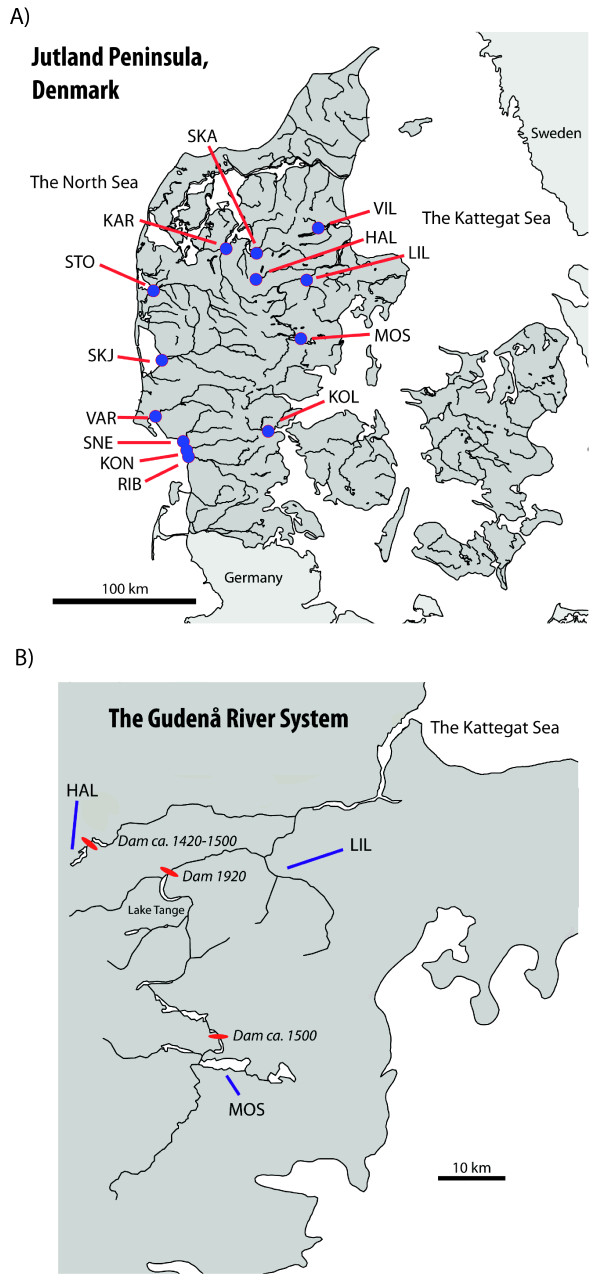
**Sampled localities. A)** Map showing the location of sampled brown trout populations in Denmark. **B)** Location of LIL, HAL and MOS in the Gudenå River system, Denmark.

Most salmonid fish species form local, genetically differentiated populations, many of which are locally adapted [29,30]. This is also the case for the brown trout (*Salmo trutta*), where several recent studies based on genetic markers and outlier scans [31-33], transcriptomics [34] and quantitative genetics experiments [35-38] have provided evidence for local adaptation. Specifically, in a common garden experiment a lake-dwelling brown trout population from Lake Hald, Central Jutland, Denmark (Figure 1) showed evidence for being adapted to higher water temperatures during incubation of eggs and larvae as compared to other populations [37]. In a second study, both the Lake Hald and another lake-dwelling trout population from Lake Mossø (Figure 1) showed several outlier loci in microsatellite DNA outlier scans encompassing anadromous, landlocked and hatchery trout populations [32].

Both Lake Hald and Lake Mossø are part of the same river system, the Gudenå River (Figure 1). There are no natural barriers to migration within the system and historically anadromous trout would have access to all parts of the river. However, over the past ca. 800 years impassable dams have increasingly been established. During the Medieval water mills, often owned and managed by monasteries, were established at many rivers for manufacturing of textiles and grinding of flour. Whereas the first types of water mills built from ca. 1200 may not have completely and permanently blocked the rivers, from ca. 1400 new types of mills became established that involved permanent damming and created impassable barriers [39]. Lake Hald and Lake Mossø became isolated from the headwaters of the Gudenå River system during that time and must be assumed to have been inaccessible to anadromous brown trout from the downstream part of the river system ever since. Hence, the question arises as to how historical anthropogenic fragmentation has affected the genetic structure, demographic parameters and potentially adaptive divergence of these populations. Did the lake trout populations diverge naturally thousands of years ago, potentially as far back in time as the end of the last Glaciation ca. 12,000 years bp? Or is habitat fragmentation due to establishment of dams the major factor shaping the current genetic population structure? What are the genetic consequences of several centuries of reproductive isolation of the populations?

In the present study we analyze variation at 44 microsatellite loci in brown trout populations from Lake Hald, Lake Mossø and anadromous trout from the lower part of the Gudenå River (the Lilleå River tributary). For reference, we additionally include 10 other anadromous populations and two hatchery strains that have been used for stocking in the region. Using individual Bayesian clustering [40] we first assess if stocking has significantly affected the current Gudenå River populations and genetic structure. Next, using analyses based on Approximate Bayesian Computation (ABC) [41] and an Isolation-with-migration model [42] we estimate divergence time between anadromous trout from the lower Gudenå River and the two lake populations. If the populations already were significantly reproductively isolated through natural processes thousands of years ago, we expect this to be reflected in the divergence time estimates. Conversely, if anthropogenic fragmentation is the primary determinant shaping the genetic structure of the populations, we expect estimates of divergence time to coincide with the establishment of dams ca. 500–800 years ago. Finally, based on results from the same analyses along with estimates of effective population size based on a linkage disequilibrium method [43] we assess the impact of dams on demographic parameters of the populations and discuss the consequences for extant and future adaptive responses.

## Methods

### Study populations

Lake Hald (in the following abbreviated HAL); see Figure 1) covers an area of 3.3 km^2^ and is inhabited by brown trout that spawn in the tributaries. The outlet is Non Mølleå River, the Danish name of which indicates that nuns from the nearby Asmild Monastery maintained a water mill at the river. Damming of the outlet and isolation of HAL from the Gudenå River system is assumed to have taken place sometime in the period from 1400 to 1500, although older mills have been constructed even earlier. Stocking of adult hatchery strain trout directly in the lake took place in the 1970s-1980s, but a study based on allozymes suggested little if any genetic impact [44].

Lake Mossø (in the following abbreviated MOS; see Figure 1) covers an area of 16.9 km^2^ and is also inhabited by brown trout that spawn in the tributaries. The nearby Øm Monastery established a water mill and permanent dam at Rye Mølle downstream of the lake around 1500. However, remnants have been found of older water mills constructed 100–200 years earlier. There has been limited stocking of hatchery strain trout in some of the tributaries, and transplants from the lower part of the Gudenå River could potentially also have affected the genetic composition.The Lilleå River (see Figure 1; abbreviated LIL) is a major tributary of the lower Gudenå River. Access to the Gudenå River for spawning anadromous trout was significantly reduced after construction of the Tange hydropower plant (see Figure 1) in 1920. The majority of the present anadromous trout population of the Gudenå River now spawn in LIL downstream of the hydropower plant, and we considered trout from this river as representative of the trout population downstream HAL and MOS. The population could potentially be affected by stocked hatchery strain trout, in particular indirectly due to stocking elsewhere in the system.

To investigate if stocked hatchery strain trout genetically affected the populations, which could influence estimation of divergence time and demographic parameters, we included two hatchery strains in the analyses, Vork Hatchery (VOR) and Hårkær Hatchery (HAR). Stocking with hatchery strain trout has not been permitted in Denmark since 2003, but was wide-spread previously. More than 80% of all stocked fish were derived from four strains that share a common history and have been shown to exhibit close genetic relationships [45]. VOR and HAR belong to this group of strains, which was originally founded by wild spawners from the Vejle and Kolding River on the East coast of Jutland.

Finally, to assess possible sources of gene flow into the LIL population and to provide a comparison to estimates of demographic parameters and to populations known to be introgressed by stocked hatchery strain trout, we included samples from 6 anadromous trout populations on the Jutland West coast (Storå River, STO; Skjern River, SKJ; Varde River, VAR; Sneum River, SNE; Kongeå River, KON; Ribe River, RIB), two populations from the Limfjord (Karup River, KAR; Skals River, SKA) and two populations from the Jutland East coast (Villestrup River, VIL; Kolding River, KOL) (see Figure 1). The West coast rivers, particularly SKJ and VAR, have previously been found to be strongly admixed with hatchery strain trout [46]. In contrast, KAR has been heavily stocked with hatchery trout, but nevertheless shows very low admixture [47]. SKA has not been stocked and to our knowledge the same applies to the VIL and KOL populations. However, KOL is known to be a main source for the founding of the quantitatively most important hatchery strains, including VOR and HAR, and could for that reason be expected to show close genetic relationships to these strains.

All samples were collected by electrofishing conducted from 1999–2009 and encompassed multiple cohorts. Electrofishing was conducted by technical staff at the Danish Institute for Fisheries Research (now National Institute of Aquatic Resources, technical University of Denmark), who had all necessary permits from the Danish Ministry of Food, Agriculture and Fisheries for electrofishing and sampling of tissue and followed all required regulations. The fish were anaesthetized and a small piece of adipose fin was removed and stored in 96% ethanol, following which the fish were released. Sampled localities, sample abbreviations, ecotypes (lake-dwelling, anadromous, hatchery trout) and sample sizes are summarized in Table 1.

**Table 1:** Information on sampled populations

**Population**	**Abbreviation**	**Geographical region**	**Ecotype**	**Sample size**
Lake Hald	HAL	Eastern Jutland, Kattegat Sea (Gudenå River system)	Lake-dwelling	32
Lake Mossø	MOS	Eastern Jutland, Kattegat Sea (Gudenå River system)	Lake-dwelling	32
Lilleå River	LIL	Eastern Jutland, Kattegat Sea (Gudenå River system)	Anadromous	32
Storå River	STO	Western Jutland, North Sea	Anadromous	34
Skjern River	SKJ	Western Jutland, North Sea	Anadromous	53
Varde River	VAR	Western Jutland, North Sea	Anadromous	36
Sneum River	SNE	Western Jutland, North Sea	Anadromous	35
Kongeå River	KON	Western Jutland, North Sea	Anadromous	33
Ribe River	RIB	Western Jutland, North Sea	Anadromous	31
Skals River	SKA	Central Jutland, Limfjord	Anadromous	32
Karup River	KAR	Central Jutland, Limfjord	Anadromous	32
Villestrup River	VIL	Eastern Jutland, Kattegat Sea	Anadromous	32
Kolding River	KOL	Eastern Jutland, Kattegat Sea	Anadromous	32
Hårkær Hatchery	HAR	Hatchery	Hatchery strain	35
Vork Hatchery	VOR	Hatchery	Hatchery strain	34

### Molecular markers

The study was based on a subset of the data set from a previous publication [32]. This paper analyzed 74 microsatellite markers and used outlier scans and landscape genomics approaches for detecting loci under possible selection. For the present study we omitted loci that were previously found to be outliers in terms of genetic differentiation, showed significant association with environmental parameters, were known to be linked to functional loci, and/or showed distributions of allele sizes that would indicate strong deviation from a stepwise mutation pattern. In total, we analyzed 44 loci, which are listed in Additional file 1: Table S1. For technical details on DNA extraction and molecular analyses we refer to a previous publication [32].

### Analysis of genetic variation and differentiation

Exact tests for deviations from Hardy-Weinberg equilibrium, observed (H_O_) and expected (H_e_) heterozygosity and allelic richness was quantified with FSTAT 2.9.1 [48]. Genetic differentiation between populations was quantified with an estimator of *F*_*ST*_[49] and significance tested by permuting alleles (10^4^ times) among populations using MSA 4.05 [50].

### Bayesian cluster analysis

Bayesian clustering implemented in STRUCTURE 2.3.4 [51,52] was used for estimating the number of populations/groups represented by the sampled individuals (*k*) and for estimating individual and population-level admixture proportions. The analysis assumed correlated allele frequencies and was furthermore based on the LOCPRIOR model [40], where information on the sample origin of individuals was used as prior information. For estimating the most likely *k* we conducted runs assuming *k* of 1 through 16. Each run consisted of a burn-in of 10^5^ MCMC steps, followed by 2×10^5^ steps. Ten replicates were conducted for each *k*. We plotted the probability of the data [(P(D)] and the *ad hoc* statistic Δ*K* (Evanno *et al.* 2005) which measures the steepest increase of the probability of *k*, using STRUCTURE HARVESTER [53]. CLUMPP [54] was used to find the consensus configuration based on the ten replicates for a given *k*, implementing the LargeKGreedy algorithm. Finally, membership proportions of individuals and samples to the identified clusters were visualized using DISTRUCT [55].

### Estimation of contemporary effective population size

We estimated the contemporary effective population size of all populations using the linkage disequilibrium (or more precisely gametic phase disequilibrium) method [43,56]. For this purpose we used the software LDNE [57] and excluded alleles occurring at frequencies below 0.05. Given that the samples analyzed included multiple age classes, we assume that the estimates are closer to representing N_e_ (the effective population size per generation) than N_b_ (the effective population size per cohort).

We also tested for recent bottlenecks in MOS, HAL and LIL using the method and software BOTTLENECK [58,59] and assuming a two-phase mutation model with 90% stepwise mutations [60].

### Estimation of divergence time and demographic parameters

We used Approximate Bayesian Computation (ABC) as implemented in DIYABC 2.0 [41] to assess posterior likelihoods of divergence time between LIL and HAL and between LIL and MOS, along with effective population sizes. It should be noted that DIYABC assumes that no gene flow occurs after populations have split. We assumed a generation time of 3.5 years [61]. We used wide and flat priors of *N*_*e*_ for all populations [50; 10,000] and for divergence time, *t* (30; 3,000 generations, corresponding to 105 and 10,500 years, respectively). We assumed a generalized stepwise mutation model [62] with a uniform prior distribution of mean mutation rate from 10^-4^ to 10^-3^, a prior distribution of individual locus mutation rates from 10^-5^ to 10^-2^ following a Gamma distribution with mean determined by the mean mutation rate across loci. The number of repeats per mutational event across loci was assumed to follow a geometric distribution with a uniform prior for the parameter P ranging from 0.1 to 0.6, whereas P for individual loci followed a Gamma distribution with mean determined by mean across loci and prior ranging from 10^-2^ to 9 × 10^-1^. A number of summary statistics can be chosen for estimating posterior distributions of parameters, some of which are, however, partly redundant. We followed the approach of a previous study [63] and conducted three different sets of analyses for each scenario based on combinations of summary statistics found to be useful in previous studies; 1) mean number of alleles across loci within populations, mean expected heterozygosity within populations, mean value of *M*[64] within populations, *F*_*ST*_ between populations and across loci, and index of individual assignment [65] across populations [66]; 2) mean number of alleles across loci, mean expected heterozygosity, mean allele size variance within populations within populations, and the same statistics across populations [67]; 3) *F*_*ST*_ between pairs of populations, mean individual assignment likelihoods of individuals collected in one of a pair of populations and assigned to the other [68], the mean number of alleles per locus between pairs of populations, mean expected heterozygosity between pairs of populations and mean variance of allele size [69] between pairs of populations [70].

We pre-evaluated each scenario and set of priors by performing 10^4^ simulations, conducting a Principal Component Analysis (PCA) based on the summary statistics and checking if the observed data set and the cloud of simulated data were congruent. The analyses were based on simulating 10^6^ data sets, and the posterior distribution of parameters was estimated using the logit approach based on the 10^4^ (1%) data sets closest to the observed data. Following estimation of parameters we checked the model by simulating 10^3^ data sets based on the predictive posterior distribution of parameters but using a new set of summary statistics, and conducting a PCA based on the summary statistics of these simulated data sets along with the 10^4^ data sets simulated based on the prior distribution (see above) and the summary statistics from the observed data. These model checks were conducted by switching summary statistics among analyses. Hence, for analyses based on summary statistics set a) we checked the model using summary statistics set b), for the analyses using set b) we checked the model using set c), and for the analyses using set c) we checked the model using set a).

We further estimated divergence time, effective population sizes and gene flow using the Bayesian, coalescence and Markov Chain Monte Carlo (MCMC) based method IMa [42]. The method is based on an isolation-with-gene-flow model, where a single population back in time splits into two, which are subsequently connected by some gene flow. The following parameters are estimated: *t*, the point in time when the ancestral population split into two, *q*_*A*_, the effective population size in the ancestral population prior to splitting; *q*_*1*_ and *q*_*2*,_ the effective population size of population 1 and 2 after divergence; *m*_*1*_ and *m*_*2*_, the migration rate from population 2 into population 1 and from population 1 into population 2. The parameters are scaled by mutation rate. To provide unscaled parameters we assumed the microsatellite mutation rate estimated using DIYABC (see above). Based on initial trial runs we found that a heated chain approach encompassing 30 chains (parameters g1 = 0.7 and g2 = 0.4), an initial burn-in of 2 × 10^6^ MCMC steps followed by 2 × 10^6^ steps yielded convergence. We assumed a generation time of 3.5 years (see above) and the following upper bounds on scaled priors: q1 = q2 = 10, qA = 100, m1 = m2 = 175 and t = 2. We conducted three replicate runs for each population pair.

## Results

### Genetic variation and differentiation

Summary statistics for all loci in all populations (expected and observed heterozygosity, allelic richness, tests for Hardy-Weinberg equilibrium) are listed in Additional file 2: Table S2. Pairwise *F*_*ST*_ ranged from 0.002 (between the neighbouring populations RIB and KON) to 0.078 between HAL and the hatchery strain HAR. All pairwise *F*_*ST*_ estimates were statistically significant except for that between RIB and KON. The two lake populations HAL and MOS were significantly differentiated from all other populations with *F*_*ST*_ ranging from 0.025 to 0.062. Differentiation between HAL and the downstream anadromous LIL population within the Gudenå River system was 0.033, whereas *F*_*ST*_ between MOS and LIL was 0.037. Finally, *F*_*ST*_ between HAL and MOS was 0.053 (Additional file 3: Table S3).

### Bayesian cluster analysis

Analysis of the STRUCTURE output showed that Δ*K* was highest for *k* = 2, but with additional peaks for *k* = 4, 7 and 12 (Additional file 4: Figure S1). At *k* = 2 one cluster was prevalent (>90%) in the two hatchery strains, VOR and HAR, whereas the other cluster was prevalent (>90%) in KAR, SKA, MOS and HAL. The other populations showed significant proportions of both clusters (data not shown). Assuming *k* = 7 provided the most detailed separation of populations (see below), whereas assuming *k* = 12 did not provide further biologically meaningful separation (data not shown).

**Figure 2:**
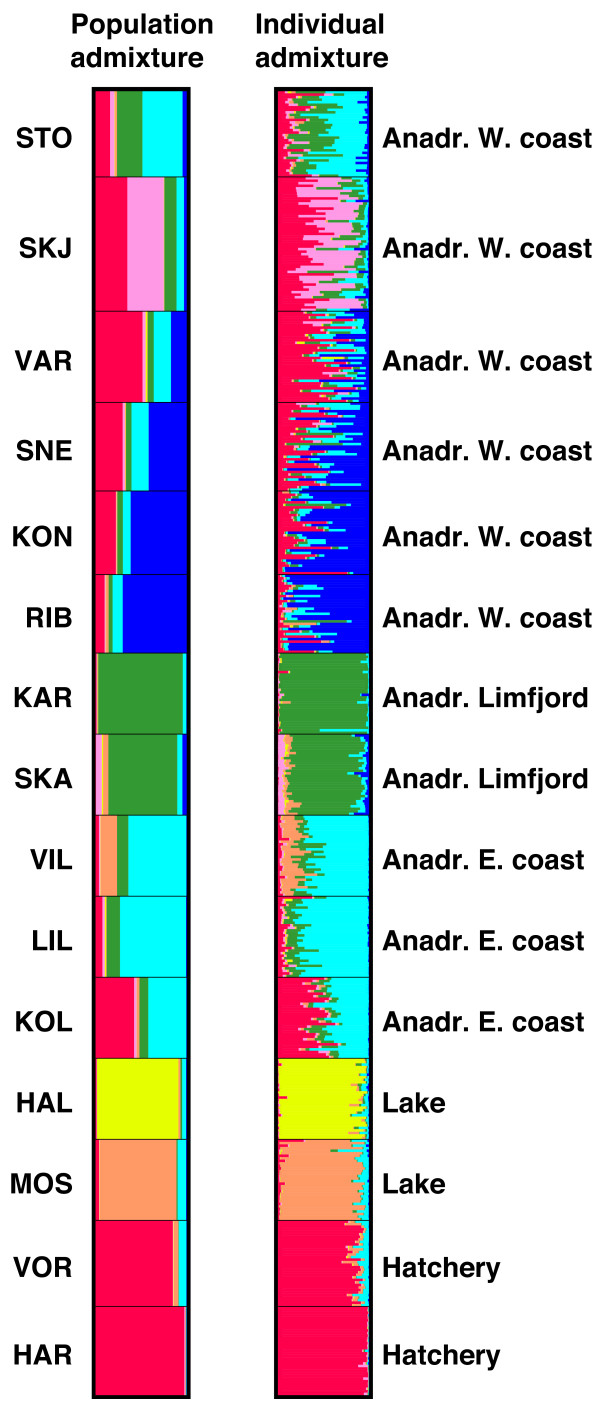
**Admixture proportions estimated using STRUCTURE 2.3.4 **[51]**,**[52]** and assuming*k*** **= 7.** The left panel shows population level admixture proportions whereas the right panel shows individual admixture proportions.

At *k* = 7, the hatchery strains VOR and HAR were characterized primarily by the “red” cluster in Figure 2, which also occurred at high frequency in the West coast populations, consistent with previous findings of admixture with hatchery strain trout [46]. The cluster was virtually absent from KAR, SKA, VIL, HAL and MOS, but was present at low frequency (0.075) in LIL and high frequency (0.423) in KOL. This could reflect a common population history, as the two hatchery strains were founded by trout from the two East coast populations KOL and the neighbouring Vejle River, or it could represent admixture due to stocking. Comparison of population and individual level cluster membership proportions (Figure 2) showed a distinct pattern for the stocked West coast populations, where some individuals showed strong admixture proportions of the “red” hatchery-specific cluster, whereas other were virtually non-admixed. In contrast, all individuals in LIL and KOL showed approximately equal proportions of the “red” cluster, indicating that a common population history rather than admixture underlies the results. We therefore assume that LIL is not admixed with hatchery trout and is representative of indigenous anadromous Gudenå River trout.HAL and MOS were characterized by two different distinct clusters (“orange” and “yellow”) whereas the two Limfjord populations KAR and SKA were characterized by a “green” cluster (Figure 2). The southern populations on the Jutland West coast (SNE, KON, RIB) showed high proportions of a “dark blue” cluster that was near absent in other populations. Finally, a “light blue” cluster was found in high proportion in the East coast populations VIL, LIL and KOL and to a smaller extent in some of the West coast populations. In total, the results suggest that the most likely sources of gene flow into LIL encompass other East coast populations and that the gene pool of this population, along with MOS and HAL are unlikely to be strongly affected by stocked hatchery strain trout.

### Contemporary effective population size

Estimates of contemporary effective population size (*N*_*e*_) using LDNE generally ranged from ca. 250 to > 1,000 in the anadromous trout populations, the exception being SKA, where the point estimate of Ne was 74 (Table 2). In RIB, *N*_*e*_ was too high to be estimated (denoted by ∞, meaning that sampling variance exceeded the signal from drift), but the lower 95% confidence interval was 480. In four anadromous trout populations (including RIB) the upper 95% confidence interval could not be determined. Specifically for LIL, representing anadromous trout from the lower Gudenå River system, *N*_*e*_ was 288. A comparable *N*_*e*_ estimate was obtained for MOS (252), whereas the estimate for HAL was lower (153).

**Table 2:** **Effective population size (*N***_***e***_**) estimates, obtained using the linkage disequilibrium method**[43]**implemented in the software LDNE**[57]

**Population**	**N**_**e**_**(95% CI)**
LIL	288 (183–647)
HAL	153 (115–227)
MOS	252 (163–533)
STO	244 (166–445)
SKJ	259 (198–370)
VAR	245 (174–407)
SNE	429 (242–1706)
KON	1317 (358-∞)
RIB	∞ (480-∞)
KAR	537 (257-∞)
SKA	74 (63–89)
VIL	369 (209–1401)
KOL	771 (307-∞)
VOR	91 (75–113)
HAR	248 (167–469)

The BOTTLENECK tests [58] provided no evidence for recent bottlenecks in neither LIL, HAL nor MOS.

### Divergence time and demographic parameters

The analyses using DIYABC yielded point estimates of mean mutation rate ranging from 2.90 × 10^-4^ to 3.85 × 10^-4^ (Table 3). Estimates of divergence time for LIL-HAL ranged from 602 to 742 years, assuming different sets of summary statistics, whereas for LIL-MOS it ranged from 599 to 641 years, thus providing strong support for divergence caused by the establishment of water mills and associated impassable dams during the Medieval. Estimates of *N*_*e*_ generated by DIYABC were substantially higher than those obtained using LDNE. Hence, point estimates of *N*_*e*_ in LIL ranged from 5790 to 7470 across different analyses, in HAL it ranged from 1980–3470 and in MOS from 3420–4070 (Table 3).

**Table 3:** **Summary of estimates of effective population size (*N***_***e***_**), divergence time and mutation rate (95% confidence intervals in parentheses) estimated using DIYABC**[41], based on different sets of summary statistics as detailed in Methods

**Populations**	**Summary statistics**	**N**_**e**_**LIL**	**N**_**e**_**HAL**	**N**_**e**_**MOS**	**Divergence time LIL-HAL (years)**	**Divergence time LIL-MOS (years)**	**Mutation rate**
LIL-MOS	Set 1	7040 (3160–9630)		3420 (922–8020)		641 (258–1236)	2.90×10^-4^ (1.63×10^-4^-6.92×10^-4^)
LIL-MOS	Set 2	5790 (2160–9430)		3670 (1170–7420)		620 (254–1386)	3.85×10^-4^ (2.33×10^-4^-8.12×10^-4^)
LIL-MOS	Set 3	5930 (2160–9460)		4070 (1180–8760)		599 (217–1246)	3.19×10^-4^ (1.76×10^-4^-7.35×10^-4^)
LIL-HAL	Set 1	7470 (3540–9730)	1980 (490–5820)		602 (245–1120)		3.02×10^-4^ (1.68×10^-4^-7.33×10^-4^)
LIL-HAL	Set 2	6830 (3010–9640)	2900 (843–6510)		742 (287–1715)		3.71×10^-4^ (2.25×10^-4^-7.95×10^-4^)
LIL-HAL	Set 3	6800 (2920–9570)	3470 (968–8360)		686 (238–1425)		3.18×10^-4^ (1.75×10^-4^-7.39×10^-4^)

**Figure 3:**
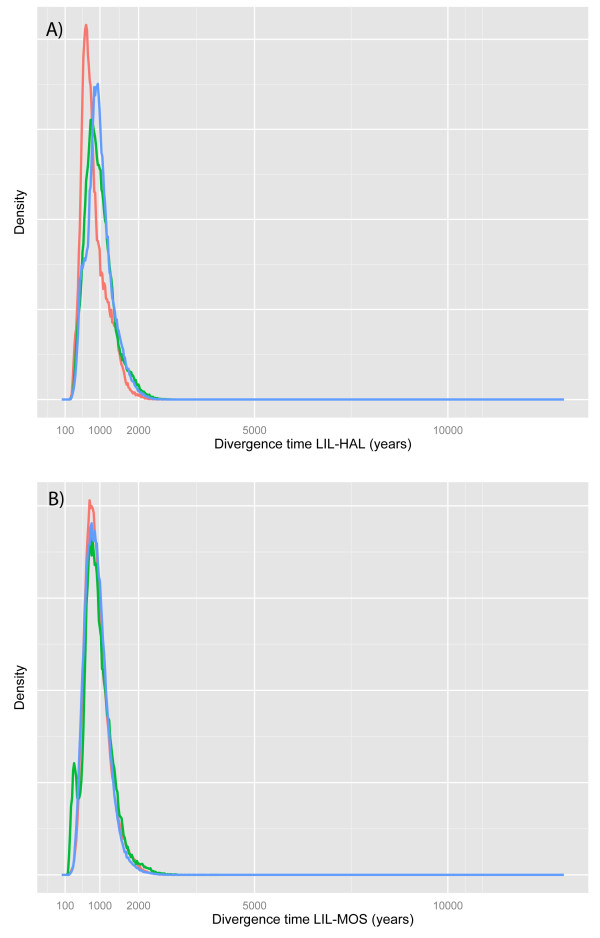
**Probability density plots of divergence time estimates obtained using IMa **[42]**. A)** Divergence time between LIL and HAL. **B)** Divergence time between LIL and MOS. The results from three replicate runs are shown, indicated by different colours.

For the IMa analyses we assumed a mutation rate of 3.0 × 10^-4^ corresponding to the estimates provided by DIYABC. The results showed highly consistent outcomes across the three replicate runs for LIL-HAL and LIL-MOS, both concerning estimates of divergence time (Figure 3) and effective population size and migration rate (Table 4). The mean divergence time across the three runs was 789 years for LIL-HAL and 696 years for LIL-MOS (Table 4), corresponding well to the estimates obtained using DIYABC. There was virtually no statistical support for divergence having occurred naturally further back in time (>1,500 years; Figure 3). Estimates of *N*_*e*_ of the two isolated lake populations MOS and HAL were lower than for the downstream LIL population (Table 4). Average modes across three runs yielded estimates of 304 in MOS and 369 in HAL, whereas average mode of *N*_*e*_ in LIL across six runs was 1585. Hence, the estimates were higher than those obtained using LDNE, but considerably lower than suggested by DIYABC. Estimates of *N*_*e*_ of the ancestral population prior to divergence were high, generally on the order of 40,000 (Table 4). Gene flow estimates from LIL and upstream above dams to HAL and MOS were, as expected, virtually zero. Also, there was limited support for gene flow downstream from HAL to LIL, whereas low but non-negligible gene flow from MOS to LIL was indicated (Table 4).

**Table 4:** **Summary of results from three runs of IMa**[42]**with different starting points for each of the population pairs LIL-HAL and LIL-MOS**

**Population pair**	**Run number**	***t***	***q***_***1***_	***q***_***2***_	***q***_***A***_	***m***_***1***_ ***(LIL to HAL/MOS)***	***m***_***2***_ ***(HAL/MOS to LIL)***
LIL-HAL	a	641 (339–1366)	1496 (695–2403)	497 (187–695)	40625 (34125–50041)	0.000 (0.000-0.008)	0.003 (0.000-0.015)
	b	804 (385–1525)	1588 (895–2496)	313 (196–678)	41542 (33559–51041)	0.000 (0.000-0.006)	0.004 (0.002-0.013)
	c	922 (431–1528)	1688 (954–2602)	296 (188–671)	42292 (33958–51708)	0.000 (0.000-0.006	0.004 (0.002-0.014)
LIL-MOS	a	688 (361–1271)	1588 (1004–2313)	321 (171–504)	39376 (32874–48042)	0.000 (0.000-0.002)	0.011 (0.006-0.020)
	b	712 (269–1317)	1529 (895–2346)	287 (146–537)	39874 (32042–49208)	0.000 (0.000-0.002)	0.012 (0.004-0.021)
	c	687 (360–1225)	1622 (1012–2287)	304 (170–506)	40625 (33542–48707)	0.000 (0.000-0.002)	0.011 (0.006-0.020)

*t* Denotes divergence time (in years) between the two populations assuming a standard mutation rate of 5.56×10^-4^, whereas *t*_*est_mut*_ denotes divergence time (in years) assuming a mutation rate of 3.00×10^-4^, reflecting the mutation rate estimates obtained using DIYABC. *q*_*1*_ denotes effective population size of LIL, *q*_*2*_ effective population size of HAL or MOS and *q*_*A*_ effective population size of the ancestral source population prior to divergence. *m*_*1*_ denotes migration rate per generation from MOS or HAL into LIL, and *m*_*2*_ denotes migration rate from LIL to HAL or MOS. The analyses are based on coalescence, but to increase clarity estimates of *m*_*1*_ and *m*_*2*_ are reported in forward-time. All estimates consist of modes and 90% credible intervals (in parentheses).

## Discussion

Divergence time estimates obtained using two different methods based on Approximate Bayesian Computation (DIYABC) and an Isolation-with-migration model (IMa) led us to reject the hypothesis that divergence of HAL and MOS from the anadromous LIL population is natural and occurred several thousand years ago. Instead, divergence time coincided remarkably well with establishment of water mills and impassable dams in the Medieval from ca. 1200 to 1500. There was less congruence regarding the demographic impact of the establishment of dams, as DIYABC consistently provided higher estimates of effective population sizes than did IMa and estimates based on the linkage disequilibrium method (LDNE).

Bayesian cluster analysis showed that the two lake populations HAL and MOS were genetically distinct, whereas the anadromous trout populations showed a regional genetic structure, reflecting ongoing gene flow and isolation-by-distance, as documented in other studies [32,46]. Importantly, these results also showed a minimal genetic contribution by stocked trout to the focal populations LIL, MOS and HAL, which could otherwise complicate estimation of divergence time and demographic parameters.

In the following, we first discuss the reliability of the estimates provided by DIYABC and IMa, and subsequently the impact of the impassable dams on genetic population structure and adaptive divergence.

### Reliability of estimates

The conclusion that the two lake populations diverged from anadromous trout due to habitat fragmentation during the Medieval relies on the results using the methods IMa [42] and DIYABC [41]. In both cases we used wide and flat priors that should not strongly influence posterior distributions. The mutation rate assumed, however, is a factor of uncertainty. For the IMa analyses we assumed a rate of 3.0 × 10^-4^, estimated using DIYABC. This is quite close to estimates of mutation rate at dinucleotide loci in e.g. humans of 2.73 × 10^-4^[71] and 5.56 × 10^-4^ in common carp (*Cyprinus carpio*) [72]. Hence, the mutation rate assumed must be considered realistic, and it would require a much lower mutation rate (2.01 × 10^-5^) to change the estimate of divergence time for LIL-HAL from 789 years to, say, 12,000 years, coinciding with the end of the last Glaciation.

Whereas the congruence of results from different replicated runs of IMa suggests good convergence, a potential problem consists in the simplified model assumed, where LIL exchanges migrants with either HAL or MOS. In reality, both populations could simultaneously contribute to gene flow, but even more importantly LIL would be subject to gene flow from other anadromous populations. We are unable to resolve the magnitude of this potential problem, but we note that the results obtained generally make sense. For both lake populations the estimate of gene flow from LIL was virtually zero, as would be expected given the problems of passing the dams upstream. In contrast, there was a signal of downstream gene flow into LIL from MOS. Gene flow in this direction is certainly a realistic possibility, as fish can pass the dams downstream by simply being flushed with the current.

*N*_*e*_ estimates of HAL and MOS obtained using IMa were of the same order of magnitude, albeit higher than estimates obtained independently using LDNE. In contrast, estimates of *N*_*e*_ in LIL obtained by IMa were more than 5 times times higher than those derived from LDNE. Although recent bottlenecks could explain this result, there was no evidence for this to have taken place. Alternatively, the result could reflect gene flow from other anadromous populations into LIL, thereby inflating historical *N*_*e*_ estimates (as in IMa) compared to contemporary estimates (as in LDNE) that pertain to the current or only a few generations back in time. The complexities of estimating *N*_*e*_ in populations showing some degree of geographical continuity are increasingly acknowledged [73], and we tentatively suggest that the *N*_*e*_ estimate in LIL based on IMa should be interpreted more broadly as encompassing LIL and neighboring populations contributing to gene flow.

The DIYABC analyses appeared robust towards the choice of summary statistics, as evidenced by similar outcomes of the different analyses. However, unlike IMa it is assumed that there is no gene flow following splitting of populations. In the present case, this is probably not a major problem, as IMa suggested low gene flow between LIL and HAL/MOS, and divergence time estimates were quite similar between methods. Effective population size estimates, however, differed strongly between IMa and DIYABC, with the latter providing 5–10 times higher estimates than IMa. In both cases the estimates are “historical”, i.e. encompassing many generations, although IMa estimates *N*_*e*_ from the ancestral population prior to divergence and *N*_*e*_ of the separate populations after divergence, the latter thereby giving more weight to the recent past (in the present case the centuries since establishment of dams). Most summary statistics applied in DIYABC, such as expected heterozygosity, *F*_*ST*_ and numbers of alleles should primarily reflect historical *N*_*e*_ over a very long time scale [74], with the exception of *M*[64] which detects recent bottlenecks. Differences of the time scale over which *N*_*e*_ is estimated could therefore explain the discrepancies of results. We emphasize the *N*_*e*_ estimates obtained by IMa as being most realistic and relevant in a conservation context; they are generally congruent with estimates of contemporary *N*_*e*_ obtained using LDNE and with estimates from Danish brown trout populations obtained using temporal methods, that for anadromous populations in Danish rivers generally range from ca. 250 to > 1,000 for large river systems [46,61].

In total, we find the estimates of divergence time obtained by IMa and DIYABC to be robust, whereas *N*_*e*_ estimates differ, probably reflecting the different time scales that they apply to.

### Impact on genetic population structure and adaptive divergence

Our results suggest that the two lake populations HAL and MOS were previously part of a coherent anadromous trout population inhabiting the Gudenå River system. The Gudenå River is the largest watershed in Denmark, and the effective population size of the ancestral trout population prior to fragmentation has presumably been high; > 1,000, given the estimates obtained from the KON and RIB populations (see Table 2 and [46]). Gene flow has occurred to and from other such populations enabling introduction of new adaptive variation and potentially both enhancing and limiting local adaptation, depending on local selection regimes and migration-drift-selection equilibrium [24,25]. Following the establishment of water mills, HAL and MOS have been disconnected from these dynamics for several centuries.

What is the primary impact of this fragmentation? Contemporary *N*_*e*_ was estimated to 153 and 252 for HAL and MOS, respectively. Although these values cannot be considered high, they should also not be cause for immediate conservation concern. Indeed, they are higher than several estimates of *N*_*e*_ in undisturbed resident brown trout populations from Sweden [75,76] that have been isolated naturally presumably for even longer time spans that the MOS and HAL populations, and are in fact comparable to *N*_*e*_ in some of the anadromous populations included in the study (Table 2). It should be considered, however, that several of these latter populations included for reference have likely declined recently, as documented by comparison of historical and contemporary samples from the populations [46].

We suggest that the most important consequence of the dams concerns local adaptation and evolutionary potential. Even if trout ascend the dams downstream and migrate to sea, they will be unable to return to spawn. This could impose strong selection against anadromy, although the response to selection depends on heritability of the trait. There are few studies available that have estimated heritability of anadromy in salmonids. However, a study based on pedigreeing a wild brook trout (*Salvelinus fontinalis*) population found heritability of life-history tactics (anadromy *versus* residency) as high as 0.52-0.56 [77]. If this is also the case in brown trout, then the 600–700 years since establishment of dams corresponding to 170–200 generations should have left ample opportunities for selective responses to occur. On the other side, a recent study of trout in HAL documented that 15% of individuals aged between 1 and 3 years and with a length exceeding 12 cm left the lake and would potentially undertake migration to the sea, whereas 40% migrated into the lake and 44% remained in the tributaries [78]. Hence, a potential for long-distance migration involving anadromy seems still to be present in the population, although it is unknown if a larger proportion of individuals would have left the lake prior to the establishment of dams.

The lack of immigration from other populations into the lakes should in the short term reduce influx of locally maladaptive alleles thereby shifting the migration-selection balance in favor of local adaptation. A common garden experiment including both LIL and HAL trout demonstrated significantly different temperature-related reaction norms for early life history traits, with HAL showing adaptation to higher incubation temperatures during winter owing to the spawning tributaries being fed by ground water [37]. Whereas this selection regime would have been similar prior to the establishment of dams, local adaptation would be expected to be reduced depending on the rate of immigration from other populations and their degree of maladaption [79].

In the long term, it is expected that reproductive isolation of the lakes combined with the relatively low effective population sizes should impose limits to the response to selection, due to the fact that loss of potentially adaptive variation is not counteracted by introduction of new variation through migration. This is all the more serious, as anthropogenic pressure increases the need for populations to adapt to environmental change [2,4,80], and specifically for brown trout it is predicted that its future distribution will become significantly reduced due to loss of suitable habitat [81]. The requirements for maintaining evolutionary potential have been expressed in the classical “500 rule” [27] (but see also [82] and [83] for discussion), stating that *N*_*e*_ should be 500 or more for new mutations to balance loss of quantitative variation by drift. This criterion is not fulfilled in HAL and MOS. Assuming no mutation, the limit to response to selection, R(∞) should be given by the expression R(∞) = 2*N*_*e*_ R(1), where R(1) denotes the initial response to selection. Moreover, the time until 50% of the response, t_50%_ is roughly equal to 1.4*N*_*e*_[84]. If we assume that HAL became isolated 700 years ago, corresponding to 200 generations and that *N*_*e*_ is 153, then t_50%_ is 214 generations, approximately equal to the number of generations that have elapsed since isolation of the population. Although this is based on simplified assumptions, it nevertheless suggests that there should still be adaptive potential within HAL and the presumably larger MOS population. Of course, different conclusions could apply to other anthropogenically isolated populations with much lower *N*_*e*_.

## Conclusions

Landscapes are presently being strongly modified by humans, but in some regions, such as large parts of Europe this is a process that has already been ongoing for centuries and even millennia. Hence, it is often difficult to ascertain that a given genetic population structure is natural rather than the result of anthropogenic modifications. In this study, we show that genetic divergence of lake-dwelling trout in two Danish lakes reflects establishment of water mills and impassable dams ca. 600 years ago rather than a natural genetic population structure. Also, the populations have historically been part of a larger system of anadromous brown trout. The reproductive isolation of the lakes is likely to have affected adaptive divergence among populations, in the short term by shifting the migration-selection balance. However, in the long term lack of gene flow combined with relatively low effective population size may compromise evolutionary potential.

The results demonstrate that caution is warranted when analyzing genetic population structure and interpreting it as “natural”. Even though anthropogenic modification of habitats has accelerated during the past century, it may nevertheless be a process that has been ongoing over a much longer time scale with significant consequences for the affected populations. Our results exemplify the use of molecular dating for assessing anthropogenic factors affecting wild populations. This is similar to other recent studies demonstrating that population decline in Borneo orang-utans (*Pongo pygmaeus*) is more recent and drastic than previously assumed [85], and conversely that Californian fishers (*Martes pennanti*) became fragmented and experienced population declines prior to European settlement [86].

### Availability of supporting data

The data set supporting the results of this article is available from Dryad: http://datadryad.org/resource/doi:10.5061/dryad.d364t at http://datadryad.org [87].

## Competing interests

The authors declare that they have no competing interests.

## Authors’ contributions

MMH and MTL conceived the study. MMH and MTL analyzed the data, with inputs and suggestions from ALF and JMP. MMH drafted the paper with contributions from MTL, ALF and JMP. All authors contributed to, read and approved the final manuscript.

## Supplementary Material

Additional file 1: Table S1 List of analyzed microsatellite loci. Click here for file

Additional file 2: Table S2 Summary table listing allelic richness, expected and observed heterozygosity and outcomes of tests for Hardy-Weinberg equilibrium at each locus in each population. Click here for file

Additional file 3: Table S3 Pairwise F_ST_ between populations Click here for file

Additional file 4: Figure S1 Assessment of the number of groups (k) represented by the sampled individuals, based on STRUCTURE [51,52] analysis of multilocus genotypes. Click here for file
