# Supplementary material for: The effects of Medieval dams on genetic divergence and demographic history in brown trout populations
Source: BMC Evol Biol. 2014 Jun 5;14:122. doi: 10.1186/1471-2148-14-122 (PMC4106231; doi:10.1186/1471-2148-14-122)
Supplement: Additional file 1: Table S1 — List of analyzed microsatellite loci. [file 1471-2148-14-122-S1.docx]

Table S1. Analyzed microsatellite loci.

| **Locus** | **Reference** |
| --- | --- |
| T3-13 | Estoup et al., (1998b) |
| SsOSL311 | Slettan et al., (1995) |
| Oneμ8 | Scribner et al., (1996) |
| Omm1073 | Rexroad et al., (2002b) |
| Ssa23NVH | Gharbi et al., (2006) |
| MST85 | Presa and Guyomard, (1996) |
| SSOSL32 | Slettan et al., (1997) |
| Ssa85 | O'Reilly et al., (1996) |
| Omm1163 | Rexroad et al., (2002b) |
| Ssa94NVH | Gharbi et al., (2006) |
| Ssa51NVH | Gharbi et al., (2006) |
| Ssa156NVH | Gharbi et al., (2006) |
| Str60 | Estoup et al., (1993) |
| Ssa73NVH | Gharbi et al., (2006) |
| Str15 | Estoup et al., (1993) |
| Ssa52NVH | Gharbi et al., (2006) |
| Ssa161NVH | Gharbi et al., (2006) |
| SSOSL438 | Slettan et al., (1996) |
| Str12INRA | Gharbi et al., (2006) |
| Ssa41NVH | Gharbi et al., (2006) |
| MST-543 | Presa and Guyomard, (1996) |
| Oneμ9 | Scribner et al., (1996) |
| Ssa87NVH | Gharbi et al., (2006) |
| Ssa71NVH | Gharbi et al., (2006) |
| Ssa54NVH | Gharbi et al., (2006) |
| Ssa26NVH | Gharbi et al., (2006) |
| Ssa39NVH | Gharbi et al., (2006) |
| Ssa7NVH | Gharbi et al., (2006) |
| Ssa24NVH | Gharbi et al., (2006) |
| BS-131 | Estoup et al., (1998b) |
| Str73 | Estoup et al., (1993) |
| Omy301UoG | Jackson et al., (1998) |
| Str2INRA | Estoup et al., (1998a) |
| SSLEEN82 | Gharbi et al., (2006) |
| One107 | Gharbi et al., (2006) |
| Ssa100NVH | Gharbi et al., (2006) |
| Ssa4DIAS | Gharbi et al., (2006) |
| OmyFGT32TUF | Gharbi et al., (2006) |
| SSOSL417 | Slettan et al., (1995) |
| Ssa63NVH | Gharbi et al., (2006) |
| Ssa207NVH | Gharbi et al., (2006) |
| Omm1116 | Rexroad et al., (2002b) |
| Ssa408UoS | Cairney et al., (2000) |
| Ssa197 | O'Reilly et al., (1996) |

References

Cairney M, Taggart JB, Høyheim B (2000). Characterization of microsatellite and minisatellite loci in Atlantic salmon (*Salmo salar* L.) and cross-species amplification in other salmonids. *Molecular Ecology*, **9:** 2175-2178.

Danzmann RG, Jackson TR, Ferguson MM (1999). Epistasis in allelic expression at upper temperature tolerance QTL in rainbow trout. *Aquaculture*, **173:** 45-58.

Estoup A, Gharbi K, SanCristobal M, Chevalet C, Haffray P, Guyomard R (1998a). Parentage assignment using microsatellites in turbot (*Scophthalmus maximus*) and rainbow trout (*Oncorhynchus mykiss*) hatchery populations. *Canadian Journal of Fisheries and Aquatic Sciences*, **55:** 715-725.

Estoup A, Presa P, Krieg F, Vaiman D, Guyomard R (1993). (CT)_n_ and (GT)_n_ microsatellites: a new class of genetic markers for *Salmo trutta* L. (brown trout). *Heredity*, **71:** 488-496.

Estoup A, Rousset F, Michalakis Y, Cornuet JM, Adriamanga M, Guyomard R (1998b). Comparative analysis of microsatellite and allozyme markers: a case study investigating microgeographic differentiation in brown trout (*Salmo trutta*). *Molecular Ecology*, **7:** 339-353.

Gharbi K, Gautier A, Danzmann RG, Gharbi S, Sakamoto T, Hoyheim B, et al. (2006). A linkage map for brown trout (*Salmo trutta*): chromosome homeologies and comparative genome organization with other salmonid fish. *Genetics*, **172:** 2405-2419.

Grimholt U, Drabløs F, Jørgensen SM, Høyheim B, Stet RJM (2002). The major histocompatibility class I locus in Atlantic salmon (*Salmo salar* L.): polymorphism, linkage analysis and protein modelling. *Immunogenetics*, **54:** 570-581.

Guo SW, Thompson EA (1992). Performing the exact test of Hardy-Weinberg proportion for multiple alleles. *Biometrics*, **48:** 361-372.

Jackson TR, Ferguson MM, Danzmann RG, Fishback AG, Ihssen PE, O'Connell M, et al. (1998). Identification of two QTL influencing upper temperature tolerance in three rainbow trout (*Oncorhynchus mykiss*) half-sib families. *Heredity*, **80:** 143-151.

McConnell SK, Oreilly P, Hamilton L, Wright JN, Bentzen P (1995). Polymorphic Microsatellite Loci from Atlantic Salmon (*Salmo Salar*): Genetic Differentiation of North American and European Populations. *Canadian Journal of Fisheries and Aquatic Sciences*, **52:** 1863-1872.

Moghadam HAR, Poissant J, Fotherby H, Haidle L, Ferguson MM, Danzmann RG (2007). Quantitative trait loci for body weight, condition factor and age at sexual maturation in Arctic charr (*Salvelinus alpinus*): comparative analysis with rainbow trout (*Oncorhynchus mykiss*) and Atlantic salmon (*Salmo salar*). *Molecular Genetics and Genomics*, **277:** 647-661.

Nichols KM, Edo AF, Wheeler PA, Thorgaard GH (2008). The Genetic Basis of Smoltification-Related Traits in *Oncorhynchus mykiss*. *Genetics*, **179:** 1559-1575.

O'Malley KG, Sakamoto T, Danzmann RG, Ferguson MM (2003). Quantitative Trait Loci for Spawning Date and Body Weight in Rainbow Trout: Testing for Conserved Effects Across Ancestrally Duplicated Chromosomes. *Journal of Heredity*, **94:** 273-284.

O'Reilly PT, Hamilton LC, McConnell SK, Wright JM (1996). Rapid analysis of genetic variation in Atlantic salmon (*Salmo salar*) by PCR multiplexing of dinucleotide and tetranucleotide microsatellites. *Canadian Journal of Fisheries and Aquatic Sciences*, **53:** 2292-2298.

Poteaux C, Bonhomme F, Berrebi P (1999). Microsatellite polymorphism and genetic impact of restocking in Mediterranean brown trout (*Salmo trutta* L.). *Heredity*, **82:** 645-653.

Presa P, Guyomard R (1996). Conservation of microsatellites in three species of salmonids. *Journal of Fish Biology*, **49:** 1326-1329.

Reid DP, Szanto A, Glebe B, Danzmann RG, Ferguson MM (2005). QTL for body weight and condition factor in Atlantic salmon (*Salmo salar*): comparative analysis with rainbow trout (*Oncorhynchus mykiss*) and Arctic charr (*Salvelinus alpinus*). *Heredity*, **94:** 166-172.

Rexroad CE, Coleman RL, Hershberger WK, Killefer J (2002a). Eighteen polymorphic microsatellite markers for rainbow trout (*Oncorhynchus mykiss*). *Animal Genetics*, **33:** 76-78.

Rexroad CE, Coleman RL, Hershberger WK, Killefer J (2002b). Rapid communication: Thirty-eight polymorphic microsatellite markers for mapping in rainbow trout. *Journal of Animal Science*, **80:** 541-542.

Rodriguez MF, LaPatra S, Williams S, Famula T, May B (2004). Genetic markers associated with resistance to infectious hematopoietic necrosis in rainbow and steelhead trout (*Oncorhynchus mykiss*) backcrosses. *Aquaculture*, **241:** 93-115.

Sakamoto T, Danzmann RG, Okamoto N, Ferguson MM, Ihssen PE (1999). Linkage analysis of quantitative trait loci associated with spawning time in rainbow trout (*Oncorhynchus mykiss*). *Aquaculture*, **173:** 33-43.

Scribner KT, Gust JR, Fields RL (1996). Isolation and characterization of novel salmon microsatellite loci: cross-species amplification and population genetic applications. *Canadian Journal of Fisheries and Aquatic Sciences*, **53:** 833-841.

Slettan A, Olsaker I, Lie O (1997). Segregation studies and linkage analysis of Atlantic salmon microsatellites using haploid genetics. *Heredity*, **78:** 620-627.

Slettan A, Olsaker I, Lie Ø (1995). Atlantic salmon, *Salmo salar*, Microsatellites at the SSOSL25, SSOSL85, SSOSL311, SSOSL417 Loci. *Animal Genetics*, **26:** 281-282.

Slettan A, Olsaker l, Lie Ø (1996). Polymorphic Atlantic salmon, *Salmo salar* L., microsatellites at the SSOSL438, SSOSL439, SSOSL444 Loci. *Animal Genetics.*, **27:** 57-58.

Somorjai IML, Danzmann RG, Ferguson MM (2003). Distribution of temperature tolerance quantitative trait loci in Arctic charr (*Salvelinus alpinus*) and inferred homologies in rainbow trout (*Oncorhynchus mykiss*). *Genetics*, **165:** 1443-1456.

Vasemägi A, Nilsson J, Primmer CR (2005). Seventy-five EST-linked Atlantic salmon (*Salmo salar* L.) microsatellite markers and their cross-amplification in five salmonid species. *Molecular Ecology Notes*, **5:** 282-288.
