# Supplementary material for: The effects of Medieval dams on genetic divergence and demographic history in brown trout populations
Source: BMC Evol Biol. 2014 Jun 5;14:122. doi: 10.1186/1471-2148-14-122 (PMC4106231; doi:10.1186/1471-2148-14-122)
Supplement: Additional file 3: Table S3 — Pairwise FST between populations [file 1471-2148-14-122-S3.docx]

Table S3. Above diagonal: Pairwise F_ST_ between populations. Below diagonal: Results of tests for the significance of F_ST_.

|  | SKJ | VAR | SNE | STO | KON | RIB | KAR | SKA | VIL | LIL | KOL | HAL | MOS | VOR | HAR |
| --- | --- | --- | --- | --- | --- | --- | --- | --- | --- | --- | --- | --- | --- | --- | --- |
| SKJ |  | 0.015 | 0.020 | 0.015 | 0.027 | 0.037 | 0.034 | 0.033 | 0.025 | 0.019 | 0.017 | 0.049 | 0.052 | 0.027 | 0.026 |
| VAR | *** |  | 0.007 | 0.010 | 0.018 | 0.027 | 0.033 | 0.026 | 0.015 | 0.014 | 0.009 | 0.042 | 0.036 | 0.012 | 0.021 |
| SNE | *** | *** |  | 0.011 | 0.009 | 0.013 | 0.034 | 0.025 | 0.019 | 0.013 | 0.015 | 0.041 | 0.034 | 0.024 | 0.035 |
| STO | *** | *** | *** |  | 0.020 | 0.029 | 0.019 | 0.016 | 0.014 | 0.012 | 0.013 | 0.041 | 0.038 | 0.027 | 0.037 |
| KON | *** | *** | *** | *** |  | 0.002 | 0.040 | 0.032 | 0.028 | 0.023 | 0.022 | 0.052 | 0.053 | 0.039 | 0.047 |
| RIB | *** | *** | *** | *** | N.S. |  | 0.050 | 0.039 | 0.035 | 0.030 | 0.035 | 0.060 | 0.054 | 0.052 | 0.061 |
| KAR | *** | *** | *** | *** | *** | *** |  | 0.020 | 0.036 | 0.025 | 0.033 | 0.048 | 0.062 | 0.056 | 0.063 |
| SKA | *** | *** | *** | *** | *** | *** | *** |  | 0.024 | 0.018 | 0.024 | 0.043 | 0.044 | 0.046 | 0.060 |
| VIL | *** | *** | *** | *** | *** | *** | *** | *** |  | 0.016 | 0.019 | 0.041 | 0.025 | 0.033 | 0.042 |
| LIL | *** | *** | *** | *** | *** | *** | *** | *** | *** |  | 0.011 | 0.033 | 0.037 | 0.033 | 0.040 |
| KOL | *** | *** | *** | *** | *** | *** | *** | *** | *** | *** |  | 0.038 | 0.039 | 0.016 | 0.026 |
| HAL | *** | *** | *** | *** | *** | *** | *** | *** | *** | *** | *** |  | 0.053 | 0.061 | 0.078 |
| MOS | *** | *** | *** | *** | *** | *** | *** | *** | *** | *** | *** | *** |  | 0.053 | 0.066 |
| VOR | *** | *** | *** | *** | *** | *** | *** | *** | *** | *** | *** | *** | *** |  | 0.022 |
| HAR | *** | *** | *** | *** | *** | *** | *** | *** | *** | *** | *** | *** | *** | *** |  |

*** p < 0.001 after false discovery rate correction.
