# Supplementary material for: The effects of Medieval dams on genetic divergence and demographic history in brown trout populations
Source: BMC Evol Biol. 2014 Jun 5;14:122. doi: 10.1186/1471-2148-14-122 (PMC4106231; doi:10.1186/1471-2148-14-122)
Supplement: Additional file 4: Figure S1 — Assessment of the number of groups (k) represented by the sampled individuals, based on STRUCTURE [51,52] analysis of multilocus genotypes. [file 1471-2148-14-122-S4.docx]

Fig. S1. Assessment of the grouping of individuals, based on STRUCTURE [[1](#_ENREF_1),[2](#_ENREF_2)] analysis of multilocus genotypes. The figure plots the *ad hoc* statistic *ΔK [*[*3*](#_ENREF_3)*]*, measuring the steepness of increase of the probability of the data representing from 1 to 16 clusters (K).


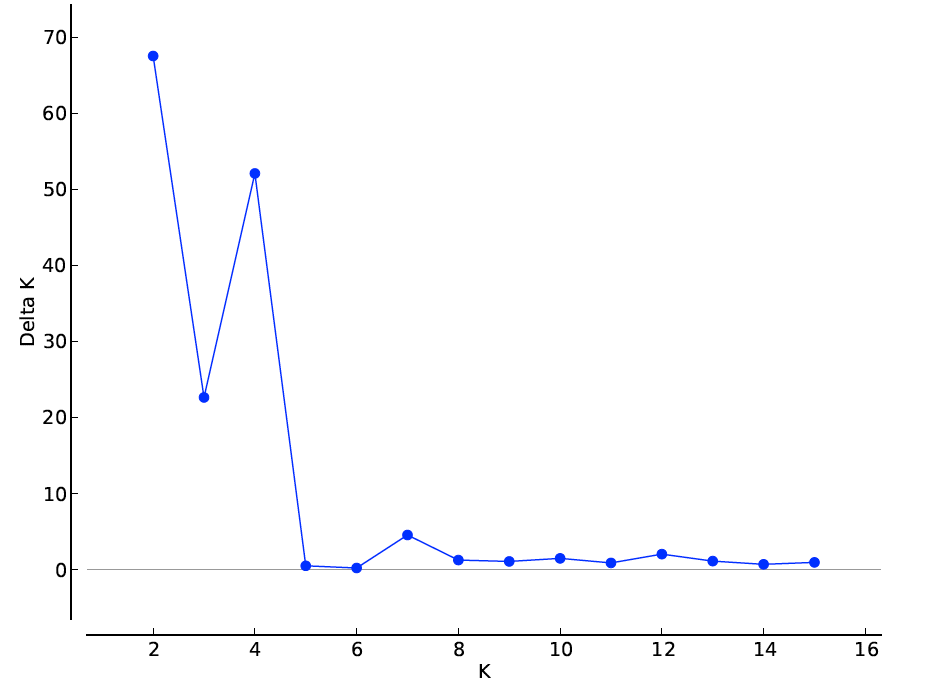


References

1. Pritchard JK, Stephens M, Donnelly P (2000) Inference of population structure using multilocus genotype data. Genetics 155: 945-959.

2. Falush D, Stephens M, Pritchard JK (2003) Inference of population structure using multilocus genotype data: linked loci and correlated allele frequencies. Genetics 164: 1567-1587.

3. Evanno G, Regnaut S, Goudet J (2005) Detecting the number of clusters of individuals using the software STRUCTURE: a simulation study. Molecular Ecology 14: 2611-2620.
